# Valvular thrombosis complicating tricuspid valve replacement: a case report

**DOI:** 10.11604/pamj.2024.49.127.45485

**Published:** 2024-12-18

**Authors:** Nugraha Teguh Palin, Achmad Lefi

**Affiliations:** 1 Department of Cardiology and Vascular Medicine, Faculty of Medicine, Airlangga University, Surabaya, Indonesia,; 2 Department of Cardiology and Vascular Medicine, Dr. Soetomo General Academic Hospital, Surabaya, Indonesia,; 3 Echocardiography Division, Department of Cardiology and Vascular Medicine, Faculty of Medicine, Airlangga University, Surabaya, Indonesia,; 4 Echocardiography Division, Department of Cardiology and Vascular Medicine, Dr. Soetomo General Academic Hospital, Surabaya, Indonesia

**Keywords:** Prosthetic valve thrombosis, anticoagulation, tricuspid valve replacement, case report

## Abstract

Valvular heart disease is cardiovascular pathology that affects over 100 million people worldwide and is associated with significant morbidity and mortality. Although tricuspid valve disease is the least common primary valve pathology, it is linked to a significantly increased mortality rate (up to 42% within 3 years). Valve thrombosis is a rare complication following mechanical valve replacement, characterized by thrombus accumulation on the valve leaflets, leading to limited valve movement, often in patients not achieving anticoagulation targets. Mechanical valve thrombosis is suspected in post-replacement patients presenting with acute or subacute symptoms. Rapid diagnosis relies on imaging modalities such as transthoracic echocardiography (TTE) and transesophageal echocardiography (TEE), computed tomography (CT), and fluoroscopy. This case report describes a 52-year-old male presenting with sudden onset dyspnea 5 days prior to hospital admission. The patient had a history of tricuspid valve replacement with a mechanical prosthesis due to tricuspid valve destruction by infective endocarditis vegetation, along with large muscular-inlet ventricular septal defect (VSD) closure and atrial septal defect (ASD) creation. Upon arrival, the patient appeared weak and dyspneic with unstable hemodynamics and was diagnosed with mechanical tricuspid valve thrombosis. Immediate valve replacement surgery was performed, but the patient unfortunately deceased 4 days postoperatively due to septic shock. We highlight the poor outcomes of prosthesis valve thrombosis and the need of prevention with regular surveillance and adequate anticoagulation strategies. The case also emphasizes the need for rapid diagnosis and management of mechanical valve thrombosis and the challenges in distinguishing it from other conditions such as pannus formation, emphasizing the importance of combining clinical and imaging criteria for accurate diagnosis and treatment.

## Introduction

Valvular heart disease is a widespread cardiovascular condition affecting over 100 million people globally and is linked to substantial morbidity and mortality. While primary valve disease of the tricuspid is the least commonly encountered, it remains associated with a high mortality rate up to 42% within three years [1].

Prosthetic valve thrombosis is a potential complication following mechanical valve replacement. It is characterized by the accumulation of thrombus on the valve leaflets, leading to restricted movement of the mechanical valve, often occurring in patients who do not achieve target anticoagulation levels. The outcome is poor, including a high risk of repeat surgery and mortality, making its prevention through adequate anticoagulation strategies crucial. Mechanical valve thrombosis should be suspected in post-replacement patients presenting with acute or subacute symptoms. Prompt diagnosis is essential in such cases and relies heavily on imaging modalities such as TTE, TEE, CT, and fluoroscopy [2].

## Patient and observation

**Patient information:** a 52-year-old male presented with sudden onset of shortness of breath, which began 5 days prior to hospital admission. The patient had undergone tricuspid valve replacement with a mechanical prosthesis 2 months earlier due to destruction of the native tricuspid valve by endocarditis vegetation. He also underwent closure of a large muscular-inlet VSD with concurrent ASD.

**Clinical findings:** upon arrival, the patient appeared weak and dyspneic, with profound hypotension and oxygen desaturation. Physical examination revealed a grade II/IV mid-diastolic murmur at the lower left parasternal border, without an audible mechanical click. There were no signs of congestion.

**Diagnostic assessment:** electrocardiography (ECG) showed right bundle branch block and signs of right ventricular hypertrophy. Chest X-ray revealed right ventricular hypertrophy and signs of pulmonary hypertension. The lung fields were free from signs of congestion or infiltrates. Sternal wires and a mechanical valve annulus were visible at the tricuspid valve site. Laboratory tests revealed that the international normalized ratio (INR) was below the therapeutic range (1.92). TTE showed the mechanical tricuspid valve in the correct position but with parameters indicative of valve dysfunction (peak velocity 2.34 m/s, mean gradient 14.4 mmHg, velocity time integral of the tricuspid valve/velocity time integral of the left ventricular outflow, VTI TV/VTI LVO: 4.33). Additionally, the VSD patch was in place without residual flow, and the ASD creation exhibited right-to-left shunting. Further TEE demonstrated immobile tricuspid valve leaflets covered by a mass suggestive of thrombus or pannus.

**Figure 1:**
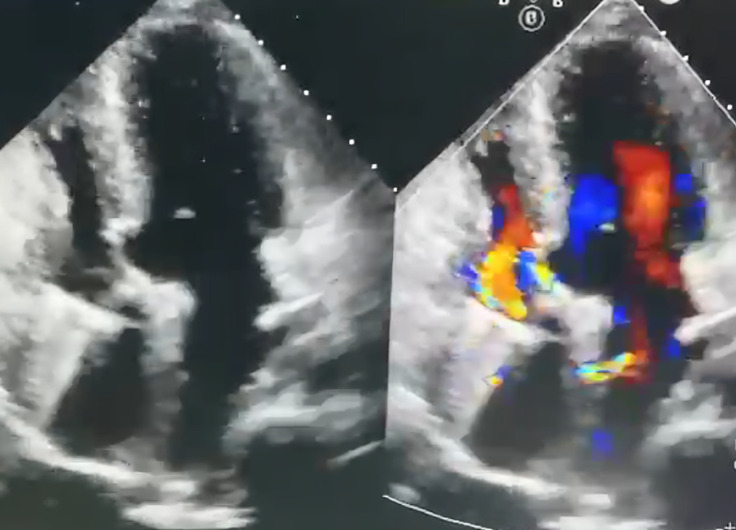
initial TTE revealed the absence of normal movement in the prosthetic valve; acoustic shadowing hindered etiological evaluation; a right-to-left shunt was observed across the ASD creation, while the VSD patch remained intact without any residual flow

**Diagnosis:** based on these findings, the patient was diagnosed with mechanical tricuspid valve thrombosis (Figure 1).

**Therapeutic interventions:** emergency valve replacement surgery was promptly performed on the patient.

**Follow-up and outcome of interventions:** postoperative evaluation with TEE showed the prosthetic valve was functioning well. However, the patient's hemodynamic status remained poor (blood pressure 87/68 mmHg with maximal IV supports). Laboratory parameters indicated progression towards septic shock (white blood cell count 18,190/µL, procalcitonin 9.7 µg/L, C-reactive protein (CRP) 6.4 mg/dL), despite aggressive antibiotic therapy and hemodynamic support. The patient was declared deceased on postoperative day 4 due to septic shock, with a total hospital stay of 7 days.

**Patient perspective:** due to the patient's passing, a patient perspective could not be provided.

**Figure 2:**
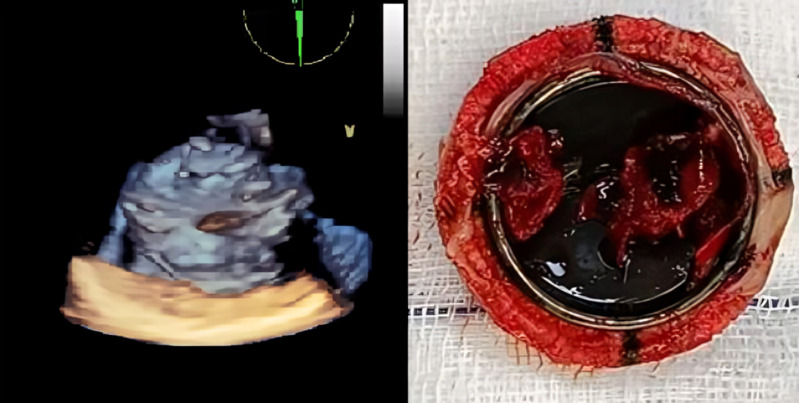
three-dimensional TEE revealed a thrombus mass covering the tricuspid valve, compared to surgically evacuated tricuspid prosthetic valve

**Informed consent:** written informed consent was obtained from the patient's family (Figure 2).

## Discussion

Tricuspid valve replacement is rarely performed because significant tricuspid valve pathology is less common. Furthermore, valve repair typically yields favorable clinical outcomes [3]. In the case we present, tricuspid valve replacement with a metal prosthesis was necessitated by the destruction of the native valve leaflets due to infective endocarditis vegetations, precluding valve repair. Mechanical valve thrombosis is one of the complications following valve replacement, caused by impaired leaflet movement and often associated with subtherapeutic vitamin K antagonist (VKA) anticoagulation [2].

Tricuspid valve thrombosis manifested as obstructive shock with severe hemodynamic instability in our case. Prosthetic valve thrombosis may also present as heart failure, thromboembolism, or hemolysis. Moreover, prosthetic valve thrombosis may be incidentally detected during routine echocardiography. Physical examination may reveal a murmur of stenosis and muffled or absent valve closure sounds [4,5].

Our initial non-invasive assessment began with TTE. Prosthetic valve dysfunction was diagnosed based on elevated transvalvular velocity or gradient, limited valve mobility, and/or mobile densities [6]. TTE can detect and quantify prosthetic valve stenosis effectively. However, the acoustic shadowing from the prosthesis limits visualization of the etiology, as encountered in our case [2]. Consequently, after initial screening with TTE, TEE was performed to better evaluate the pathological substrate of prosthetic valve dysfunction. Three-dimensional TEE may differentiate thrombus from pannus or valve vegetations. Like thrombus, vegetations typically originate in the valve annulus area and spread to the leaflets, disrupting valve coaptation [7].

Prosthetic valve thrombosis and pannus growth may be difficult to differentiate, even with TEE. Thus, a combination of clinical and imaging criteria is necessary to distinguish between these entities. Such differentiation is important because management strategies differ for each entity. Lin *et al*. described three independent predictors of mechanical valve thrombosis: increased transvalvular gradient, the presence of an occlusive mobile mass on the prosthetic valve, and an INR ≤ 2.5 [4]. These criteria were met in our case, establishing the diagnosis of valve thrombosis. CT scans and fluoroscopy were not performed because echocardiographic examination was deemed sufficient and the patient's hemodynamic condition was unstable.

Preventive strategies are essential to detect early signs of prosthetic valve dysfunction. The ESC guidelines recommend TTE monitoring after valve replacement or repair at 30 days post-surgery, at 1 year, and annually for life. TTE should also be performed if the patient develops new symptoms or if there is clinical suspicion of complications [5].

Failure to achieve therapeutic VKA dosing following prosthetic valve implantation, as seen in our patient, is a significant factor contributing to thrombosis formation. VKA, guided by INR, remains the mainstay of management in patients with mechanical prosthetic valves, while NOACs currently have no established role [5,6]. In the case we encountered, a St. Jude Medical mechanical prosthetic valve was used to replace the tricuspid valve, thus the INR was targeted to at least 3.0. The thrombogenicity of the valve in the tricuspid position is higher compared to the mitral position, allowing two possible strategies: targeting a slightly higher INR or adding low-dose ASA.

The management of mechanical heart valve (MHV) thrombosis carries significant risk, regardless of the treatment option chosen. Treatment options for prosthetic valve thrombosis include surgery, thrombolysis, and anticoagulation, depending on the patient's clinical condition [5,6]. Emergency valve replacement is recommended for obstructive prosthetic valve thrombosis in critically ill patients without contraindications for surgery, as seen in the case we encountered [5]. Surgery should also be considered for large (>10 mm) non-obstructive prosthetic valve thrombi associated with embolism or persistent despite optimal anticoagulation. After thrombus resolution and restoration of normal hemodynamics, long-term oral anticoagulation is administered to prevent recurrent valve thrombosis. Prosthetic valve thrombosis and pannus fibrotic growth can recur; hence, after hemodynamic improvement and/or valve movement restoration, serial follow-up is crucial, as residual thrombus may lead to recurrent thrombosis [2,5]. Fibrinolysis is the primary alternative option in situations where surgery cannot be performed immediately, despite the risk of bleeding and thromboembolism [6].

## Conclusion

Prosthetic valve thrombosis is a potentially fatal complication following valve replacement. Appropriate anticoagulation with VKA is essential for preventing valve thrombosis. VKA is administered to achieve a target INR based on the type of prosthetic valve, taking into account the individual patient's risk of bleeding and thrombosis. When mechanical valve thrombosis is suspected, diagnosis is confirmed using various diagnostic modalities such as TTE, TEE, and CT scans. Management with heparinization, fibrinolysis, and surgery is selected based on clinical presentation to ensure the most optimal outcome in this otherwise grave condition.
